# Mechanochemical assembly of polymer-cyclodextrin inclusion complexes *via* twin-screw extrusion for large-scale production and material reinforcement

**DOI:** 10.1039/d6cc00830e

**Published:** 2026-05-14

**Authors:** Jingchen Shi, Xiaoqing Mou, Hao Tang, Luotong Gong, Han Wu, Alexander Wahrhaftig-Lewis, Benjamin Hansberg, Stephen Schrettl, Neil R. Champness, Stefan Guldin, Lixu Yang

**Affiliations:** a School of Chemistry and Chemical Engineering, Chongqing University Chongqing 401331 China lixu.yang@cqu.edu.cn; b Department of Chemical Engineering, University College London Torrington Place London WC1E 7JE UK s.guldin@ucl.ac.uk; c School of Chemistry, University of Birmingham Edgbaston Birmingham B15 2TT UK n.champness@bham.ac.uk; d BASF SE, Process Research & Chemical Engineering, Coating & Film Processing Carl-Bosch-Strasse 38 67056 Ludwigshafen am Rhein Germany; e Technical University of Munich, Department of Life Science Engineering Gregor Mendel-Straße 4 85354 Freising Germany; f TUMCREATE, 1 CREATE Way #10-02 CREATE Tower 138602 Singapore

## Abstract

We developed a twin-screw extrusion (TSE), method to create polymer inclusion complexes (PICs) from cyclodextrin and hydrophobic polymers, namely polycaprolactone (PCL), polyethylene (PE) and polypropylene (PP). These PICs serve as effective compatibilizers to toughen polymer blends and enable bulk and surface post-polymerization functionalization of inert polymers.

## Abstract 2

We report a polymer cyclodextrin complexation method using twin-screw extrusion (TSE).

## Graphical abstract

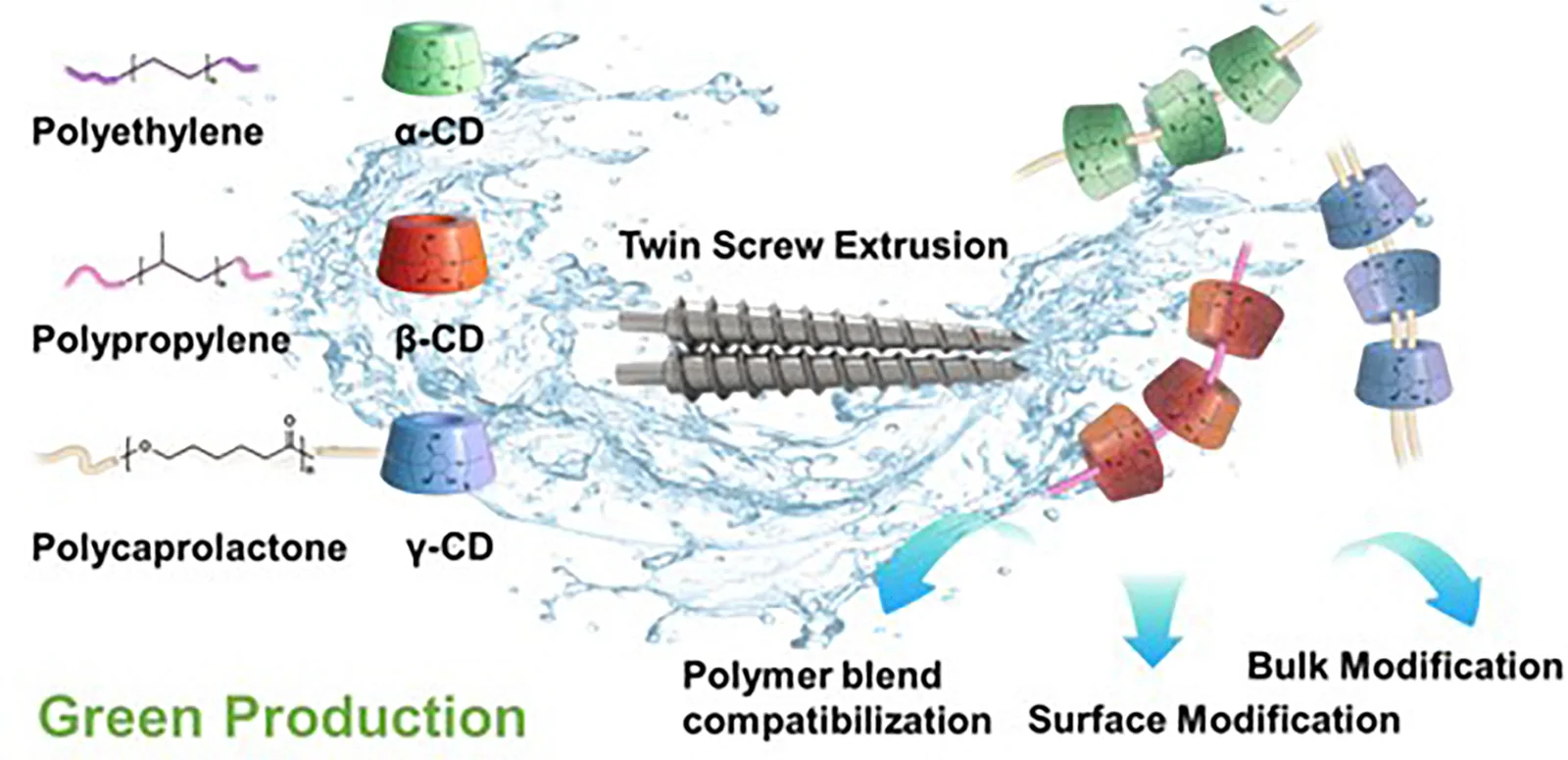

Polymers with interlocking architectures provide versatile opportunities for the development of advanced materials. One established strategy involves the synthesis of rotaxanes *via* the polymerization of interlocked monomers, while another relies on the threading of macrocycles onto pre-formed polymer chains to generate PICs, also referred to as polymer polypseudorotaxanes.^1–4^ Among the various macrocycles explored, cyclodextrins (CDs), derived from starch, are most widely employed owing to their biocompatibility, commercial availability, low toxicity and ease of chemical modification. CDs can form complexes with both hydrophilic and hydrophobic polymers, including polyethylene glycol (PEG), polypropylene glycol (PPG), polycaprolactone (PCL), oligo-ethylene, and oligo-propylene.^1,5,6^ These polymer-CD complexes can stabilize the polymer and improve biocompatibility. A less explored area is the use of PICs to improve compatibility in polymer blends through controlled coalescence from the inclusion state.^7^ PICs have consequently found use in the design of tough materials,^8^ sensors,^9^ batteries,^10–12^ drug delivery systems^13^ and other functional materials.^3^ Recently, significant effort has been directed toward integrating PIC motifs to toughen commercially relevant polymers such as PCL and polyurethane.^14,15^ The development of scalable routes to PICs is therefore a key requirement for advancing their industrial implementation.

The most common approach to preparing CD inclusion complexes is solution-based methods, typically involving the addition of guest molecules to aqueous CD solutions with optional heating or sonication (Fig. 1a).^16^ While effective for small molecules, these strategies are poorly suited to polymeric guests, particularly hydrophobic polymers. Specifically, successful complexation in water is in general limited to polymers with a molecular weight below approximately 3000 Da.^5,6,17,18^ Although water represents a green solvent and readily dissolves CDs, it is a poor solvent for hydrophobic polymers, which tend to adopt collapsed globular or coil conformations.^19^ Yet, conversion of these conformations into extended chains required for threading is thermodynamically unfavorable in such media. To overcome this limitation, large quantities of organic solvents such as chloroform, acetone or *N*,*N*-dimethylformamide are often employed to solubilize hydrophobic polymers and pre-organize polymer chains into more extended conformations.^20–23^ However, the use of organic solvents not only raises environmental concerns, but also necessitates rigorous solvent removal, particularly for applications related to food, pharmaceuticals, and cosmetics.

**Fig. 1:**
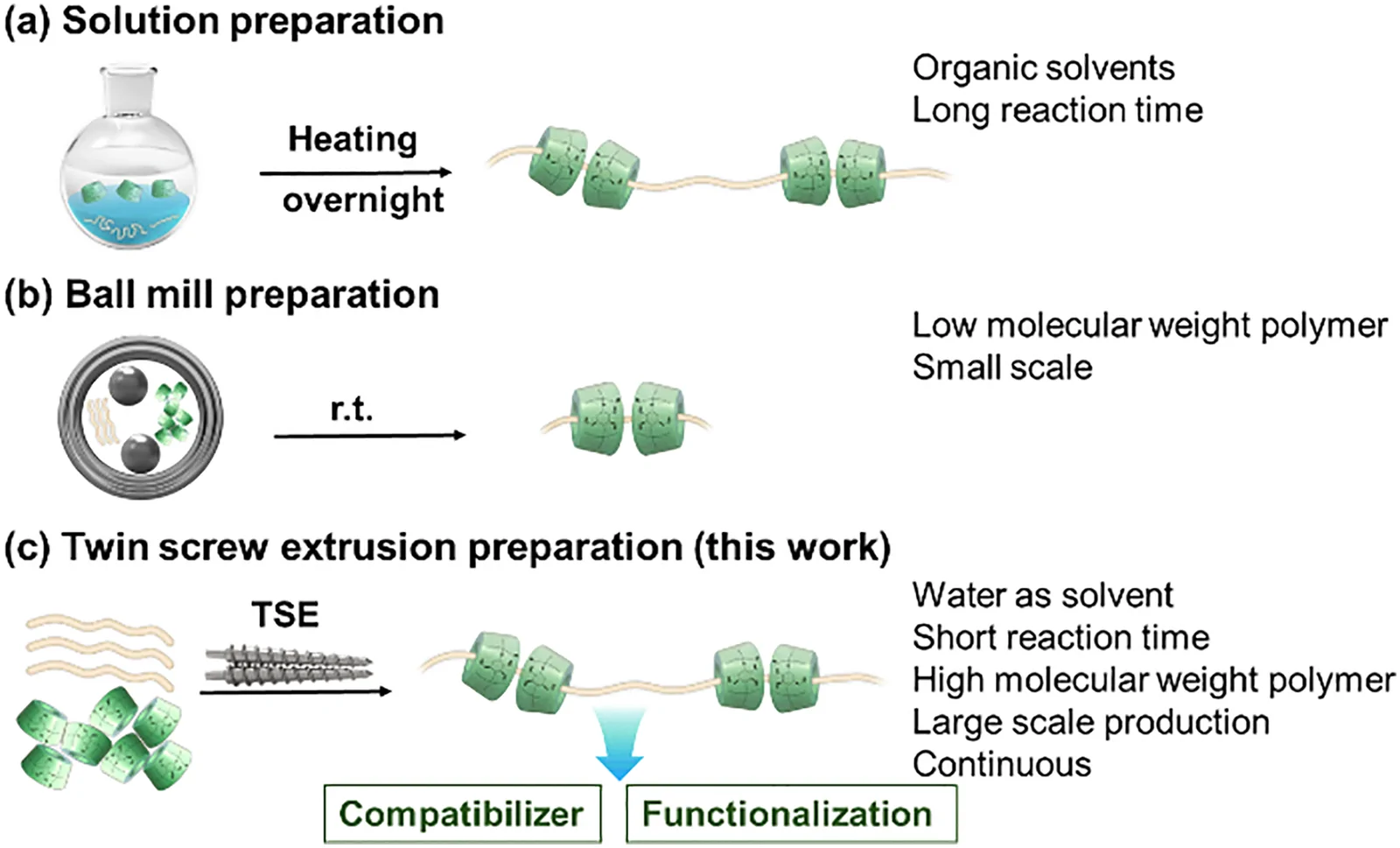
Schematic illustration of representative preparation methods for polymer-cyclodextrin inclusion complexes (PICs): (a) conventional solution-based complexation, (b) mechanochemical preparation by ball milling, and (c) mechanochemical assembly *via* twin-screw extrusion (TSE).

While the threading of hydrophilic polymers such as PEG into CDs has been well-studied, the processing of hydrophobic semi-crystalline polymer chains into CD inclusion complexes remains challenging, primarily due to their poor solvent compatibility, and limited chain mobility under ambient conditions. A very recent study by Numata *et al.* demonstrated that dynamic flow in solution can promote the piercing of PEG chains through CD cavities, overcoming unfavorable coil conformations.^24^ Independently, we developed a strategy to harness analogous dynamic forces in the more challenging regime of hydrophobic polymer melts. Here, we report a green and scalable manufacturing approach to prepare CD inclusion complexes with hydrophobic polymers using TSE, a widely employed processing technology in the food, pharmaceutical, and polymer industries (Fig. 1c).^25^ Unlike solution-based approaches that are largely restricted to hydrophilic guests or low molecular weight polymers, our approach utilizes shear flow in the melt state to process PCL, PE and PP, commodities that together account for over half of global plastic production (Scheme S1).^26^ We further demonstrate that the resulting PICs can be processed by TSE to act as effective compatibilizers in polymer blends and enable bulk and surface functionalization.

Initial attempts to prepare CD complexes with higher molecular weight PE, PP, and PCL in aqueous solution were unsuccessful, prompting us to explore mechanochemical approaches based on ball milling (Fig. 1b). Low molecular weight PE (*M*_w_*ca.* 4000 g mol^−1^, PE4000) successfully formed complexes with CD in the presence of water. However, no successful complexation was observed for higher molecular weight PE (Table S1 and S2). Similarly, attempts to form PCL-α-CD inclusion complexes by ball milling in the presence of various solvents were unsuccessful (Table S3). Whilst conventional ball-mills do not allow for external heating, TSE, another method of mechanosynthesis, offers the combined application of mechanical shear and heating. In addition, the continuous shear and extensional flow experienced by polymer chains during extrusion promotes more extended chain conformations,^27^ which are favorable for threading into CD cavities. Motivated by these considerations, we systematically investigated TSE processing parameters, including CD content, residence time, and the use of liquid additives, for the formation of PCL-α-CD inclusion complexes (Table S4).

In initial extrusion experiments, PCL pellets and α-CD were co-extruded at 70 °C with a screw speed of 80 rpm for 45 min (Table S4, entry 1). However, no evidence for inclusion complex formation was detected by powder X-ray diffraction (PXRD) under these conditions. Notably, the formation of inclusion complexes was confirmed by the appearance of characteristic channel-type CD reflections in PXRD upon addition of a small amount of water as a liquid additive (Table S4, entry 2, Fig. S1). This suggests that complexation may be promoted by releasing high energy water from CD cavities, increasing both the enthalpic and entropic contribution to complexation free energy.^28,29^ Although inclusion complexes were detected under all water-assisted extrusion conditions, the presence of free α-CD in its cage-type crystalline form was observed for several parameter sets (Table S4, entries 3–6, Fig. S1). Shorter residence times (Table S4 entry 4, Fig. S1) or higher α-CD loadings (Table S4 entry 6, Fig. S1) resulted in reduced inclusion efficiency and increased amounts of free α-CD, as evidenced by changes in the relative intensities of the characteristic PXRD peaks, likely associated with insufficient dispersion of α-CD within the PCL melt under these conditions. In summary, optimal inclusion was achieved in the presence of water, at an α-CD content of 20%, and with longer extrusion time.

With the optimized conditions, we next explored the generality of this approach for other hydrophobic polymers and CDs (Table S5, Scheme S1). The larger cavity of γ-CD has been reported to accommodate two PCL chains.^20^ As expected, PCL and γ-CD complexes were successfully prepared by TSE. Other hydrophobic polymers, PE and PP were also complexed with α-CD and β-CD, respectively. While PP has previously been reported to form inclusion complexes with both β-CD and γ-CD under solution-based conditions,^6^ only the PP-β-CD complex was formed under TSE conditions.

Inclusion complex formation was confirmed *via* PXRD (Fig. 2). Upon complexation, free CDs transit from cage-type to channel-type crystalline structures (Fig. 2a).^30^ Characteristic channel-type reflections were observed at 2*θ* = 20.0° for α-CD complexes (PCL-α-CD, PE4000-α-CD, LLDPE-α-CD; Fig. 2b, d, e),^20^ 7.5° for PCL-γ-CD (Fig. 2c),^20,23^ and 13.1°/17.9° for PP-β-CD (Fig. 2f).^31^ Conversely, water-free extruded mixtures displayed only polymer diffraction patterns and amorphous CDs. These observations further support that the emergence of channel-type CD reflections is directly associated with polymer inclusion rather than simple physical mixing. Notably, plastic polymers from commercial products also form complexes with CDs under extrusion (Fig. S2).

**Fig. 2:**
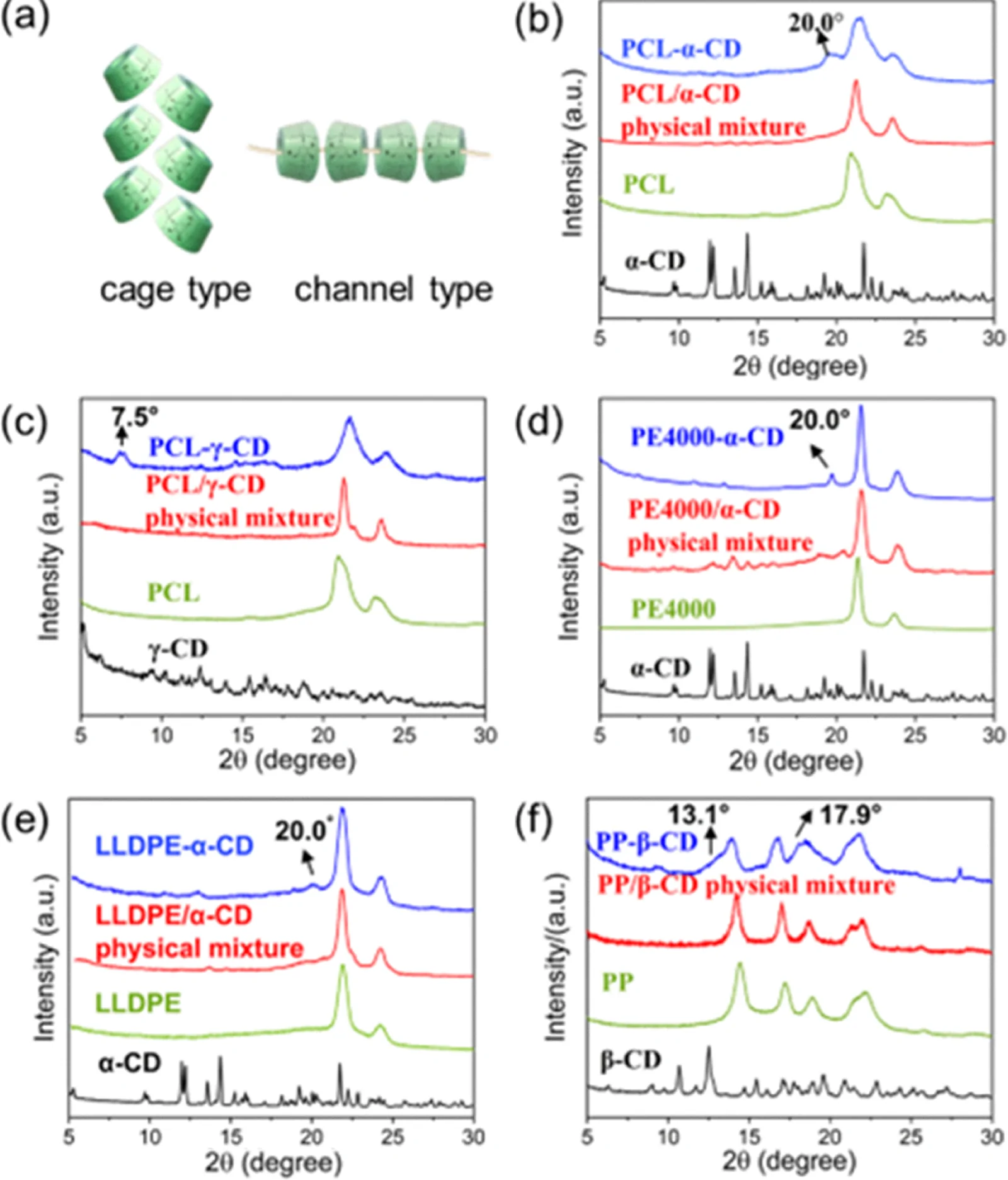
(a) Cage-type CDs reorganise into channel-type structures upon inclusion complexation, (b)–(f) Powder X-ray diffraction patterns of polymer-CD inclusion complexes, polymer CD physical mixtures, free polymers, free CDs.

Thermogravimetric analysis of the PICs showed two weight loss processes, corresponding to the thermal degradation of CDs and the polymers, respectively (Fig. S3). In all cases, the onset of polymer degradation was shifted to higher temperature compared to their neat polymers, indicating an enhanced thermal stability upon inclusion. Differential scanning calorimetry analysis reveals that complexation with CDs increases the melting temperatures of PCL, PE4000, and LLDPE, while decreasing that of PP, reflecting differences in packing and chain confinement imposed by CDs (Fig. S4).^32,33^ Tensile testing of the PIC materials PCL-α-CD, PCL-γ-CD, and LLDPE-α-CD showed reduced toughness and elongation at break relative to the parent polymers, likely due to disruption of polymer chain packing within the crystalline regions (Fig. S5).

CD clusters or polymers have been shown to improve mechanical properties of polystyrene blends *via* host–guest interactions.^34,35^ Similarly, the polymer-CD inclusion complexes reported here feature a high density of accessible hydroxyl groups acting as hydrogen-bond donors, suggesting their potential use as compatibilizers for otherwise immiscible polymer blends. As proof of concept, a polylactic acid (PLA)/PCL blend was chosen to evaluate the compatibilizing effect of PICs. While blending brittle PLA with ductile PCL improves mechanical performance, effective toughening requires controlling phase morphology and domain sizes.^36–39^ In this study, the previously reported optimal blend composition of 80/20 (PLA/PCL) was employed, and CD was added as a compatibilizer to enhance interfacial compatibility. To avoid organic solvents and simplify processing, PCL-CDs inclusion products obtained directly from TSE were used without further purification, which means free PCL, free CD and PCL-CD inclusion complex are present in the mixture. For comparison, PCL/CD physical mixtures prepared by TSE (Table S4, entry 1) were blended with PLA as a control experiment. Tensile tests were performed to study the effects of CD on mechanical properties of PLA/PCL blends (Fig. 3 and Fig. S6). Elongation at break of PLA/PCL blend was only 8.3% under our processing condition, indicating limited toughening in the absence of a compatibilizer, consistent with existing reports.^40,41^ Decrease in tensile strength and a slight increase in elongation was observed in PLA/PCL/α-CD physical mixture and PLA/PCL/γ-CD physical mixture, indicating limited interfacial reinforcement without polymer inclusion. Unlike reported CD-polystyrene blends,^34^ host–guest interactions are absent in these physical mixtures, limiting mechanical enhancement. In contrast, blends containing PLA/PCL-α-CD and PLA/PCL-γ-CD exhibited a pronounced increase in elongation at break, reaching values exceeding 110% and 150%, respectively. Both blends displayed higher tensile strength than pure PLA/PCL blend. The superior performance of PLA/PCL-γ-CD over PLA/PCL-α-CD is likely due to the slide-ring effect, as γ-CD can accommodate two PCL chains per channel. Indeed, polymer networks incorporating slide-ring motifs as cross-linking agents have previously been shown to exhibit enhanced extensibility and toughness.^4,14,42,43^ These improvements are attributed to improved interfacial adhesion mediated by hydrogen-bonding interactions (Fig. 3a), as well as energy dissipation through reversible bond dissociation during sample deformation.

**Fig. 3:**
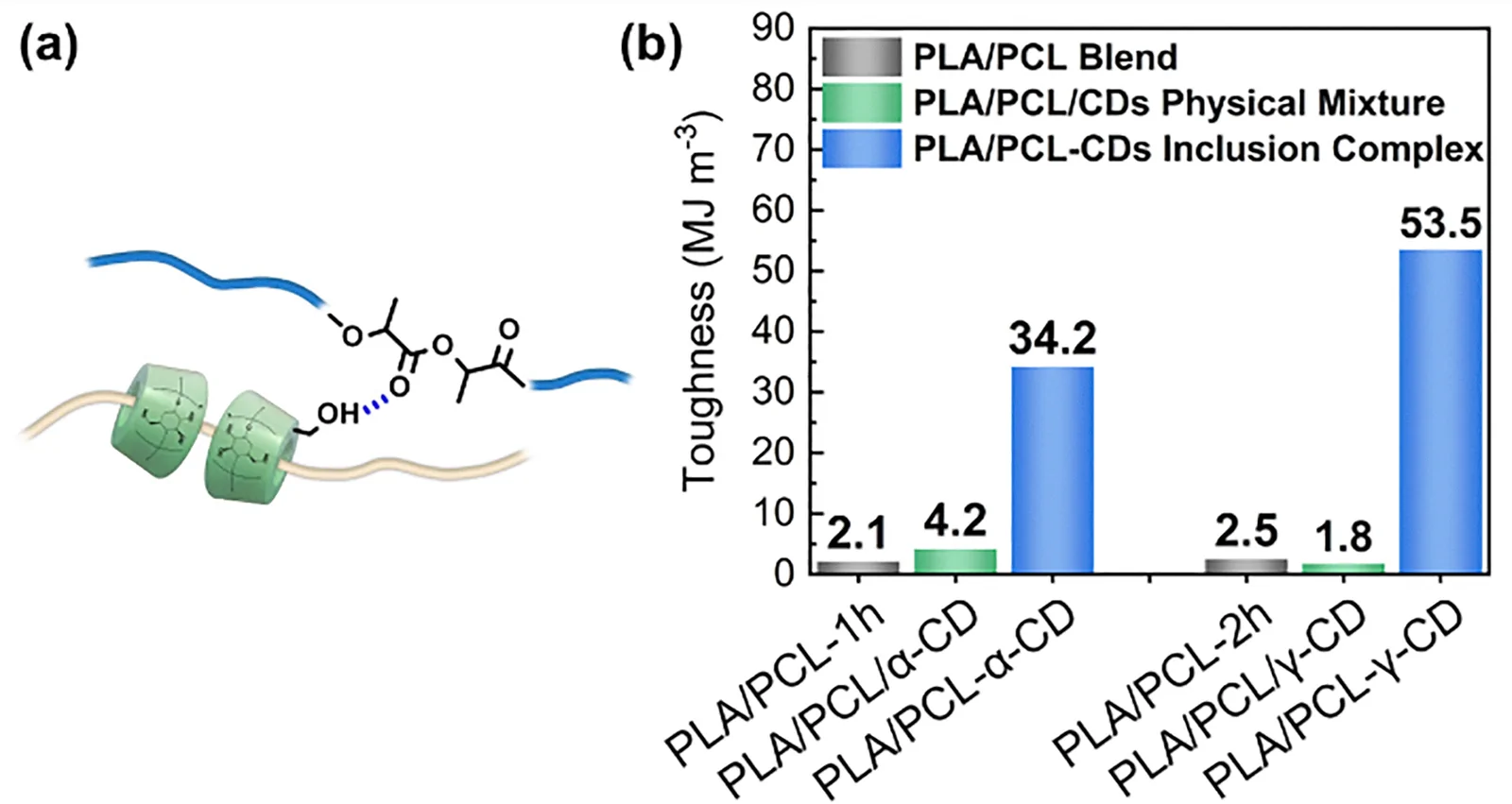
(a) Schematic illustration of H-bonding interactions between PLA (blue line) and PCL-α-CD (yellow line with green CD), other OH groups on the CD rim were omitted for clarity. (b) Comparison of the toughness of different extruded polymers.

To further evaluate the compatiblizing effects, scanning electron microscopy (SEM) was performed. For uncompatibilized PLA/PCL blend, pronounced cracks can be seen from their interfaces indicating their incompatibility (Fig. S7). Physical mixtures of PLA/PCL/α-CD exhibited rough surfaces with numerous cracks (Fig. S8a). In contrast, PCL was more uniformly dispersed within the PLA matrix in PLA/PCL-α-CD blends, and interfacial cracking was substantially suppressed (Fig. S8b). For the PLA/PCL/γ-CD physical mixture, PCL domains appeared as discrete islands within the PLA matrix, with visible cracks at the interfaces (Fig. S8c). Notably, PLA/PCL-γ-CD blend changed to a co-continuous structure with smooth surface, correlating with enhanced toughness (Fig. S8d).

To explore the generality of the compatibilization strategy, polymer-CD inclusion complexes were applied to additional immiscible polymer systems. Incorporation of LLDPE-α-CD into PLA resulted in materials with a notable increase in both tensile strength (36 MPa) and elongation at break (31%) (Fig. S9). These values are comparable to those reported for PLA/LLDPE blends compatibilized using block copolymers,^44^ highlighting the effectiveness of PICs as alternative compatibilizers. A similar trend was observed for LLDPE-α-CD/PP-β-CD blend, where improvements in mechanical performance were accompanied by more homogeneous phase morphologies. SEM analysis again confirmed enhanced interfacial adhesion in the presence of PICs (Fig. S10).

The prepared PICs offer opportunities for functionalizing inert polymers. PCL, PCL/α-CD physical mixture, and PCL-α-CD were treated with fluorescein isothiocyanate (FI) (Fig. 4a). Confocal fluorescence microscopy revealed that the surface of FI-treated PCL remained non-fluorescent, corroborating that unmodified PCL is largely inaccessible to mild surface modification and typically requires aggressive treatments like plasma or hydrolysis (Fig. 4b).^45^ PCL/α-CD physical mixture displayed only weak, sparse fluorescence, suggesting that most of the CD present at the surface was removed during preparation (Fig. 4c). In contrast, strong fluorescence appeared on the surface of FI treated PCL-α-CD sample (Fig. 4d). The well aligned lines resembled those observed in AFM, where PICs prepared by dynamic flow were bundled through CD interactions.^24^ This alignment is consistent with the extrusion-induced extension and organization of polymer chains, and further suggests that the CDs remain strongly associated with the polymer chains and are resistant to dethreading under the functionalization conditions.

**Fig. 4:**
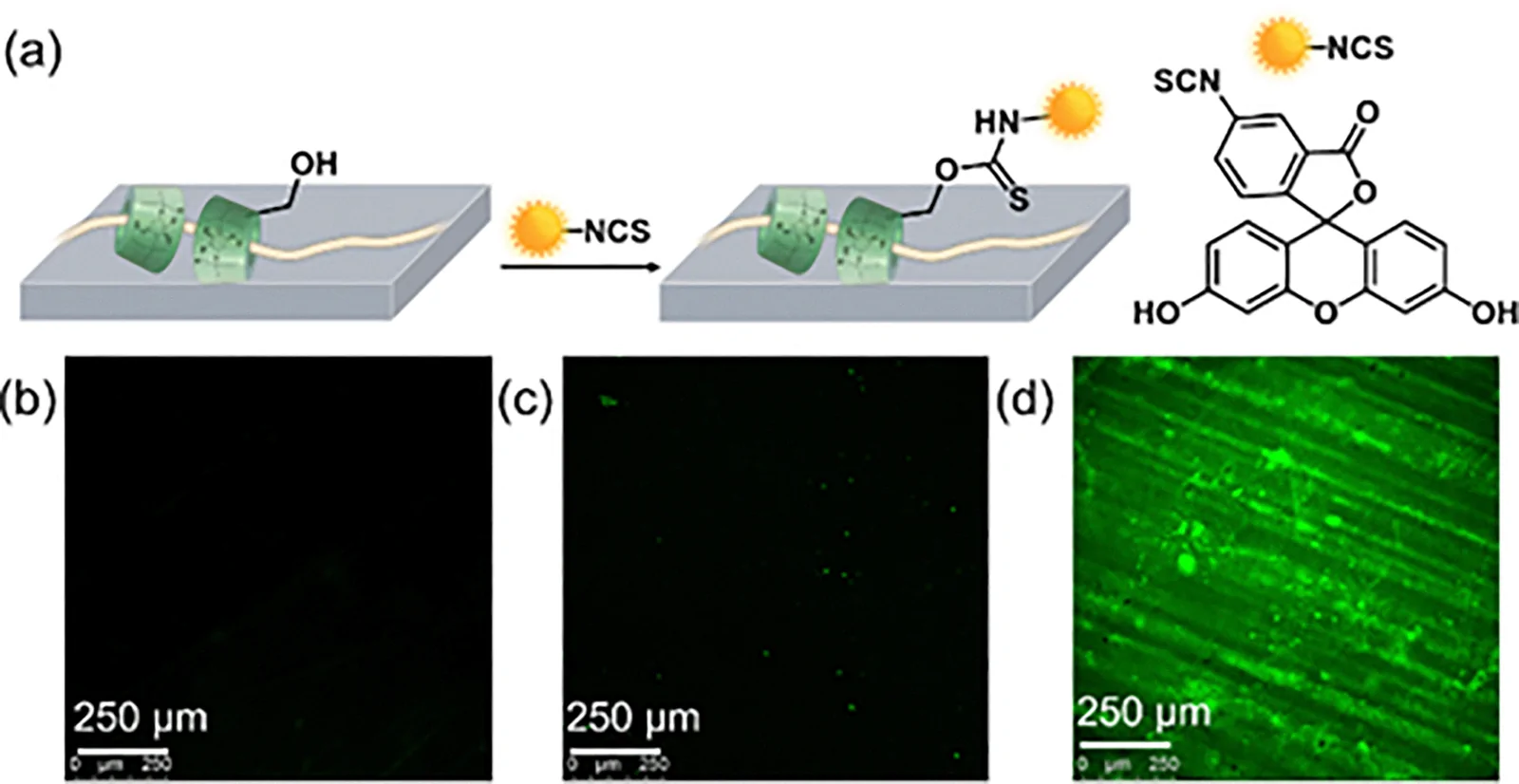
(a) PCL-α-CD film undergoes surface reaction with FI, and laser scanning confocal fluorescence microscopy images of FI-treated (b) PCL, (c) PCL/α-CD physical mixture, and (d) PCL-α-CD. Scale bar: 250 µm.

Bulk modification is frequently employed to tailor polymer mechanical properties and generate high-value materials. As a proof of concept, PCL-α-CD and PCL-γ-CD were subjected to further reactive processing with butyl isocyanate (But-NCO) *via* TSE (Fig. 5a). Fourier transform infrared (FTIR) spectroscopy confirmed urethane formation, evidenced by new absorption bands at 1616 and 1574 cm^−1^, which were notably weak in neat PCL processed under identical conditions (Fig. 5b). This difference reflects the substantially higher density of accessible hydroxyl groups in the CD-containing inclusion complexes compared to neat PCL. ^1^H NMR analysis showed that more than 50% of But-NCO was converted in the presence of PCL-α-CD and PCL-γ-CD inclusion complexes, whereas only approximately 8% conversion was observed for neat PCL under the same conditions (Fig. S10). XRD peaks at 20.0° and 7.5° were observed in PCL-α-CD/But-NCO and PCL-γ-CD/But-NCO samples respectively, implying that the CD channel structure remains intact (Fig. 5c). The stability of the PICs under reaction conditions is key for further functionalization in TSE as part of a continuous manufacturing process.

**Fig. 5:**
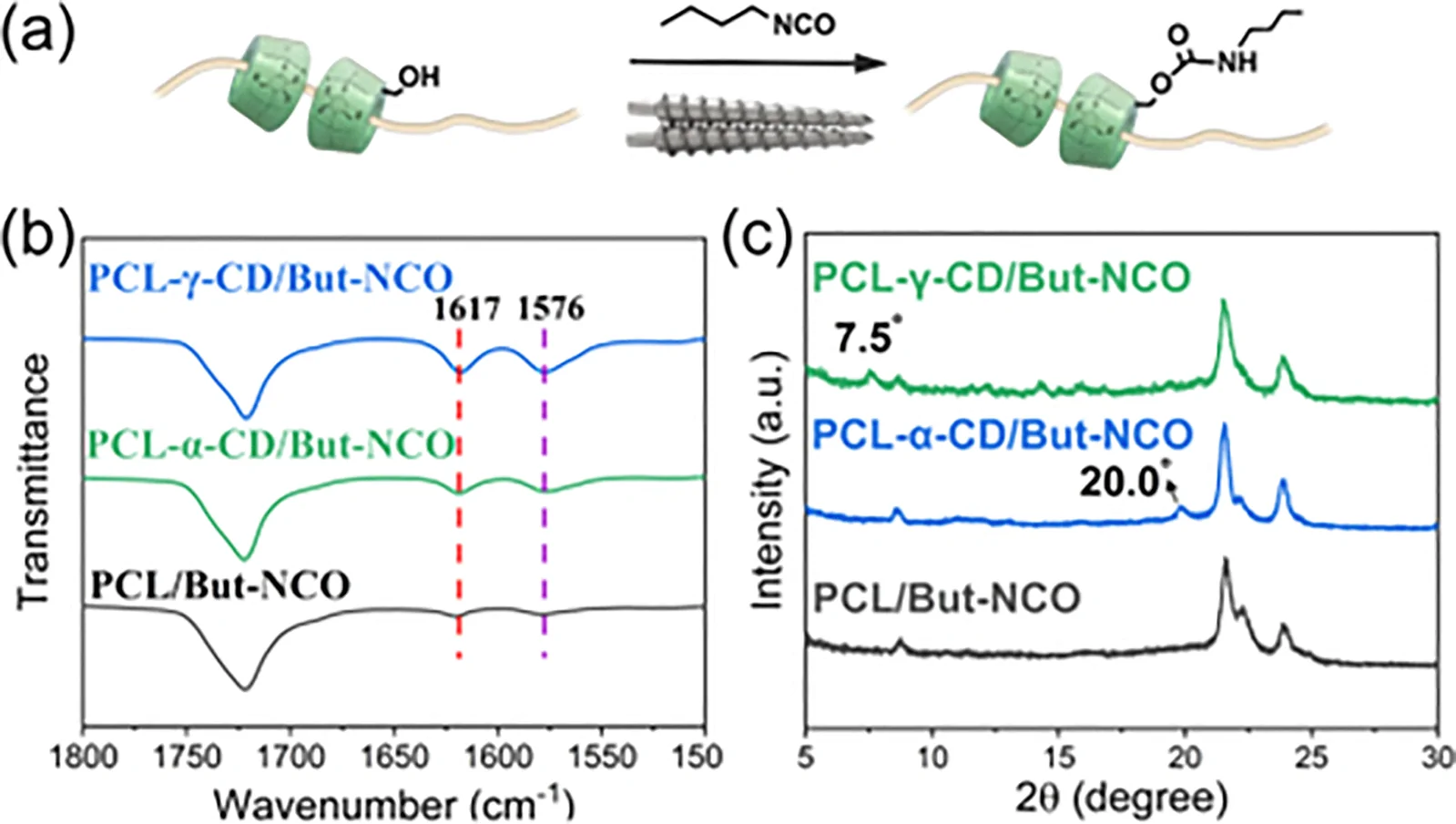
(a) Reaction of PCL-α-CD with But-NCO *via* TSE, (b) FTIR spectra and (c) PXRD patterns of PCL, PCL-α-CD and PCL-γ-CD treated with But-NCO *via* TSE.

In summary, we demonstrate twin-screw extrusion as a continuous melt-state method for preparing cyclodextrin inclusion complexes with high molecular weight hydrophobic polymers that avoids the use of organic solvents typically required in solution-based processing. The use of dynamic shear and extensional flow enables efficient threading of polymers into cyclodextrin channels, overcoming key limitations of conventional solution-based methods. This work establishes TSE as a versatile platform for the continuous production, modification, and upcycling of polymer materials based on inclusion architectures. The generality and industrial relevance of this approach offer opportunities for extending mechanochemical inclusion strategies to other macrocycles and polymer systems.

## Conflicts of interest

There are no conflicts to declare.

## Supplementary Material

CC-062-D6CC00830E-s001

## Data Availability

The supporting data has been provided as part of the supplementary information (SI). Supplementary information: Tables S1–S3, Scheme S1, PXRD patterns, TGA curves, Tensile stress–strain curves, SEM images, NMR spectra and further experimental details, see DOI: https://doi.org/10.1039/d6cc00830e.
