# Supplementary material for: Mechanochemical assembly of polymer-cyclodextrin inclusion complexes via twin-screw extrusion for large-scale production and material reinforcement
Source: Chem Commun (Camb). 2026 May 14;62(47):11860–4. doi: 10.1039/d6cc00830e (PMC13579561; doi:10.1039/d6cc00830e)
Supplement: Supplementary file 1 [file CC-062-D6CC00830E-s001.pdf]

Supplementary Information (SI) for Chemical Communications.
This journal is © The Royal Society of Chemistry 2026

Supporting Information

Mechanochemical Assembly of Polymer-Cyclodextrin Inclusion Complexes via Twin-Screw Extrusion for Large-Scale Production and Material Reinforcement

Jingchen Shi‡, Xiaoqing Mou‡, Hao Tang, Luotong Gong, Han Wu, Alexander Wahrhaftig-Lewis, Benjamin Hansberg, Stephen Schrettl, Neil R. Champness, Stefan Guldin*, Lixu Yang**

Materials

α-Cyclodextrin (α-CD), γ-cyclodextrin (γ-CD), 5-fluorescein isothiocyanate (FI), n-Butyl isocyanate (But-NCO) and polylactic acid (PLA, M_w: 110000) were purchased from Meryer Biochemical Technology Co., Ltd. (Shanghai, China). β-Cyclodextrin (β-CD) was purchased from Heowns Biochemical Technology Co., Ltd. (Tianjin, China). Polyethylene (PE4000, M_w: 4000), linear low density polyethylene (LLDPE, melt index 1.0 g/10 min (190°C/2.16kg)) and polypropylene (PP, M_w: 12000) were purchased from Sigma-Aldrich. Polycaprolactone (PCL, M_w: 65000) was purchased from Block Chemical Technology Co., Ltd. (Shanghai, China).

General

Ball mill reactions were performed using a Retsch MM400 mill using 25ml stainless steel jar and two stainless steel balls (diameter 15 mm). Twin screw extrusion was performed on experimental micro-twin-screw extruder (WLG05G produced by Shanghai Xinshuo Precision Machinery Co. Ltd.) Powder X-ray diffraction (PXRD) pattern was carried out on an Empyrean X-ray diffractometer (DY1808). The X-ray radiation used was Cu-Kα radiation with a voltage of 40 kV and a current of 40 mA, with a step size of 0.01° and a step time of 0.05 s. The scanning rate is 13 °/min, and the scanning range is from 5° to 50°. The tensile tests were conducted on an universal testing machine (M221C produced by Shanghai Hengyi Precision Instrument Co., Ltd.) with a tensile speed set at 10 mm/min, a sensor capacity of 1000 N, at room temperature and a relative humidity of approximately 52%. Samples for tensile test were prepared by hot compression under a pressure of 1.7 MPa and cut into rectangle shapes (30 mm * 2 mm * 0.5 mm). Scanning Electron Microscopy (SEM) images were taken on a Hitachi SU8010 field emission scanning electron microscope. The laser scanning confocal microscope (LSCM) images were taken on a Leica TCS SP8 at λ=488 nm. Fourier transform infrared spectrophotometry (Nicolet iS50 FITR Spectrometer produced by Thermo Fisher Scientific) was carried out using attenuated total reflection (ATR) mode in the range of 4000–400 cm^{-1}

with a resolution of 4 cm^{-1}. Thermogravimetric analysis (TGA) was performed on a SHIMADZU apparatus (DTG-60H) in the temperature range of 25 – 600 °C with a ramp rate of 10 °C/min under N_2 (flow rate of 50 mL/min). Differential scanning calorimetry (DSC) (TA Instruments, modulated DSC3+) under N_2 atmosphere at a heating rate of 10 ℃ min^{-1} and N2 flow rate 20 L min^{-1}.

Experimental

Ball mill experiments

PE4000 and α-CD were combined with 1 mL of deionized water and subjected to mechanochemical processing in a ball mill at a specified frequency (Hz) for a predetermined duration. The resulting mixture was subsequently separated by centrifugation. The floating solid, consisting of unreacted PE4000, was carefully removed and discarded. The precipitated solid was collected and washed three times with deionized water (3 × 50 mL) to remove residual unreacted α-CD.

Table S1. Ball mill reactions of PE4000 and α-CD in 1ml water with different frequencies and duration.

α-CD (mg)	PE4000 (mg)	Time (min)	Frequency (Hz)	Product (mg)
500	400	30	10	2
500	400	30	20	149
500	400	60	20	432

Table S2. Ball mill reactions (20 Hz, 60 min) of PE4000 with varying amount of α-CD.

α-CD (mg)	PE4000 (mg)	Product (mg)
100	400	0
300	400	342
500	400	432
700	400	470

Table S3. Ball mill reactions (30 Hz, 60 min) of PCL and α-CD with different solvents.

α-CD (g)	PCL (mg)	solvents
1.0	100	DCM:water (1.2 ml:1.2 ml)
0.5	100	DCM:water (1.2 ml:0.6 ml)
0.5	100	Acetone:water (1.2 ml:0.6ml)
1.0	100	DMF:water (1.2 ml:1.2 ml)

Table S4. Summary of the preparation conditions of PCL and α-CD.

Entry	α-CD content (wt %)	Mixing time (min)	Solvent	Inclusion
1	20	45	none	No
2	20	45	water	Yes
3	40	45	water	Yes
4	40	30	water	Yes
5	40	60	water	Yes
6	60	60	water	Yes

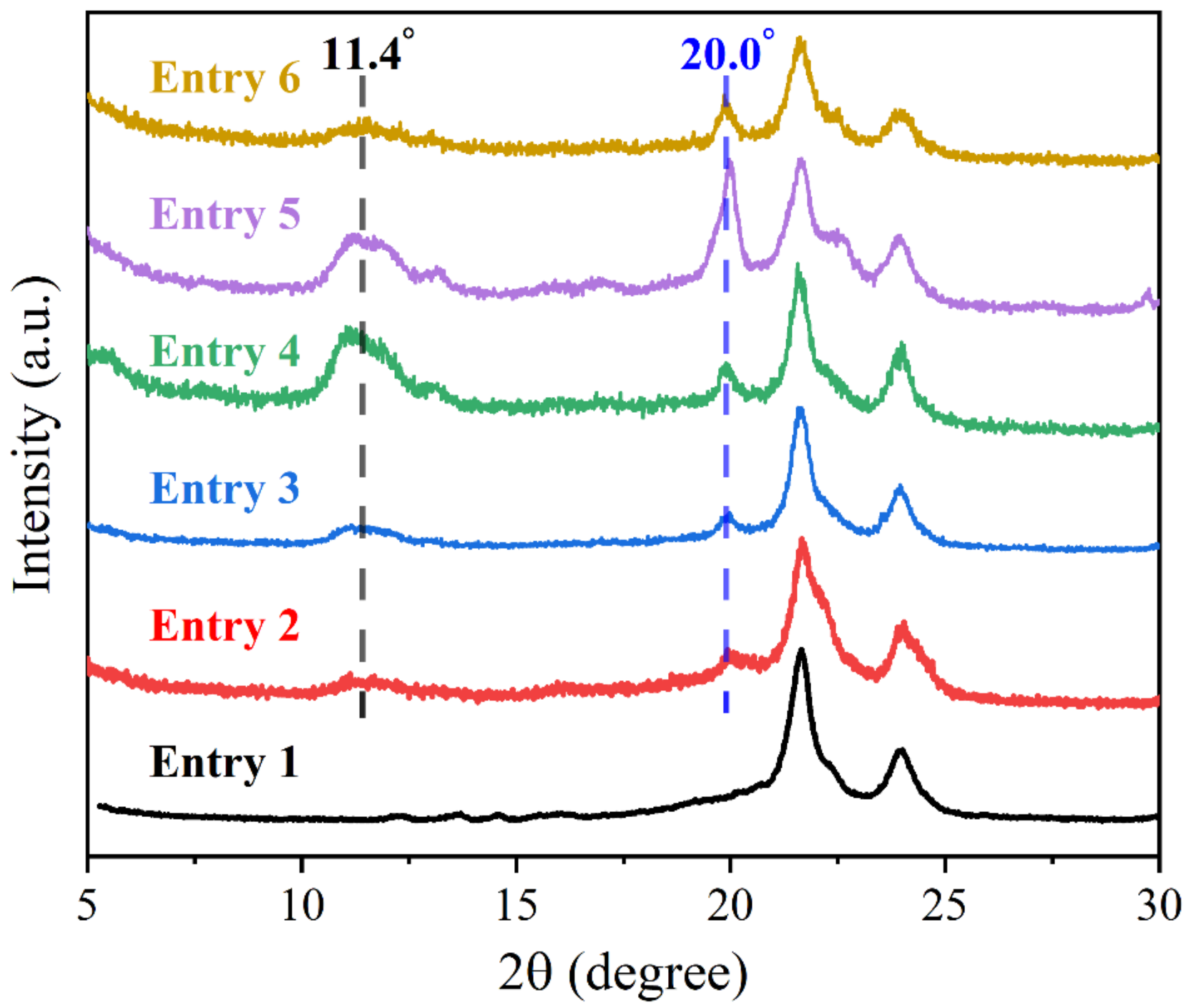

Figure S1. XRD patterns of PCL and α-CD prepared in Table S4.

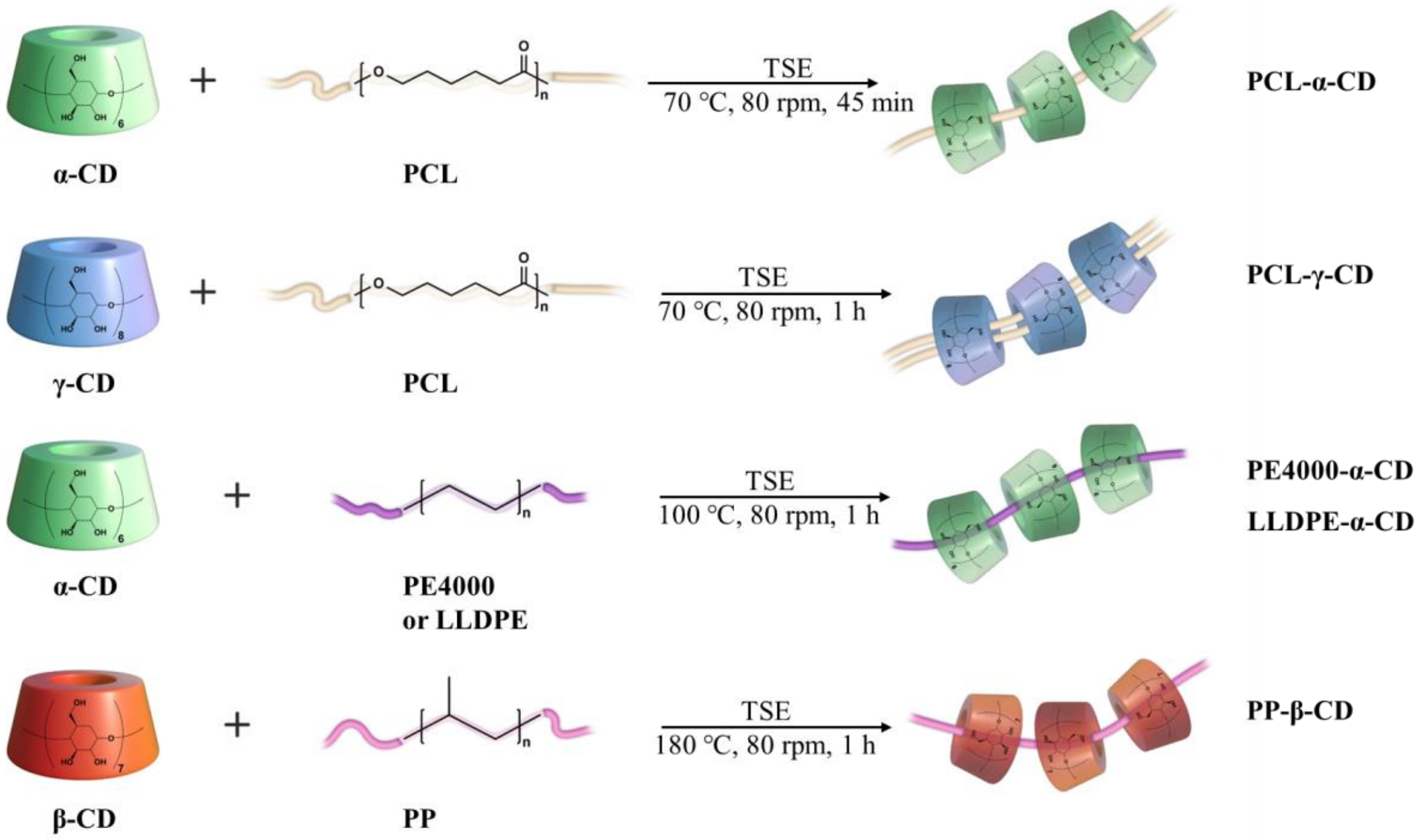

Scheme S1. Schematic representations illustrating the concept of IC formation, α-CD (green), β-CD (orange) and γ-CD (blue).

Preparation of Polymer Cyclodextrin Inclusion Complexes by TSE

1.0 g cyclodextrins (α-CD, β-CD or γ-CD) and 4.0 g polymers were mixed in a beaker with an appropriate amount of deionized water (0.6-3.2 ml) to form a paste-like mixture. The mixture was slowly introduced into the preheated twin-screw extruder at a speed of 68 rpm. After feeding, the speed was increased to 80 rpm, and the mixture was circulated in the barrel for a set duration. Then the extruder was switched from self-circulation mode to discharge mode to collect the product. The PIC samples were denoted as PCL-α-CD, PCL-γ-CD, PE4000-α-CD, LLDPE-α-CD and PP-β-CD. Control samples were subject to the same procedure without water additive. Note: crude products were subject to characterization without further purification. Polypseudorotaxanes undergo complex-uncomplex equilibrium in solution, any purification or characterization in solution do not resemble solid state process.

Preparation of Polymer Blends

PLA/PCL-α-CD blend: All materials were dried under vacuum before TSE blending. PCL-α-CD sample was cut into small pieces, and premixed with PLA pellets. PLA/PCL-α-CD of 80:20 (w/w) was added to a preheated twin-screw extruder at 180 °C with a speed of 68 rpm. After feeding, the speed was increased to 80 rpm, and the mixture was circulated in the barrel for 1

h. Then the extruder was switched from self-circulation mode to discharge mode to collect the product. PLA/PCL-γ-CD, PLA/LLDPE-α-CD were kept at the same 80:20 (w/w) proportion but was extruded for 2 h for better performance. LLDPE-α-CD/PP-β-CD was kept at 50:50 (w/w). Control samples were prepared at the same temperature, proportion and extrusion duration, respectively.

Preparation of PCL-α-CD Surface with Fluorescein Isothiocyanate (FI)

PCL-α-CD flakes (0.1 g) were prewashed thoroughly with deionized water to remove free CD on the surface, then dried under vacuum. Under dark environment, FI was dissolved in diethyl ether to obtain 10^{-6} mol/L FI stock solution. PCL-α-CD flakes were then placed in the solution and stirred for 4 hours at room temperature. The mixture was then filtered, washed with diethyl ether and dried at room temperature overnight. The samples were kept in the dark until further analysis. As a control, PCL, and a PCL/α-CD physical mixture was subject to the same treatment.

Bulk Reactions of PCL-CD with Butyl Isocyanate

PCL-α-CD or PCL-γ-CD were dried under vacuum at 70 °C overnight. The twin-screw extruder was preheated to 70 °C and purged with nitrogen gas. n-Butyl isocyanate (But-NCO, 0.4 g) and PICs (4.6 g), of which CD and But-NCO were kept at 1:4 molar ratio, were added to the twin-screw extruder at 70 °C at a speed of 68 rpm. After feeding, the speed was adjusted to 80 rpm for 3 h. Then the extruder was switched from self-circulation mode to discharge mode to collect the product. As a control, PCL was subject to the same treatment.

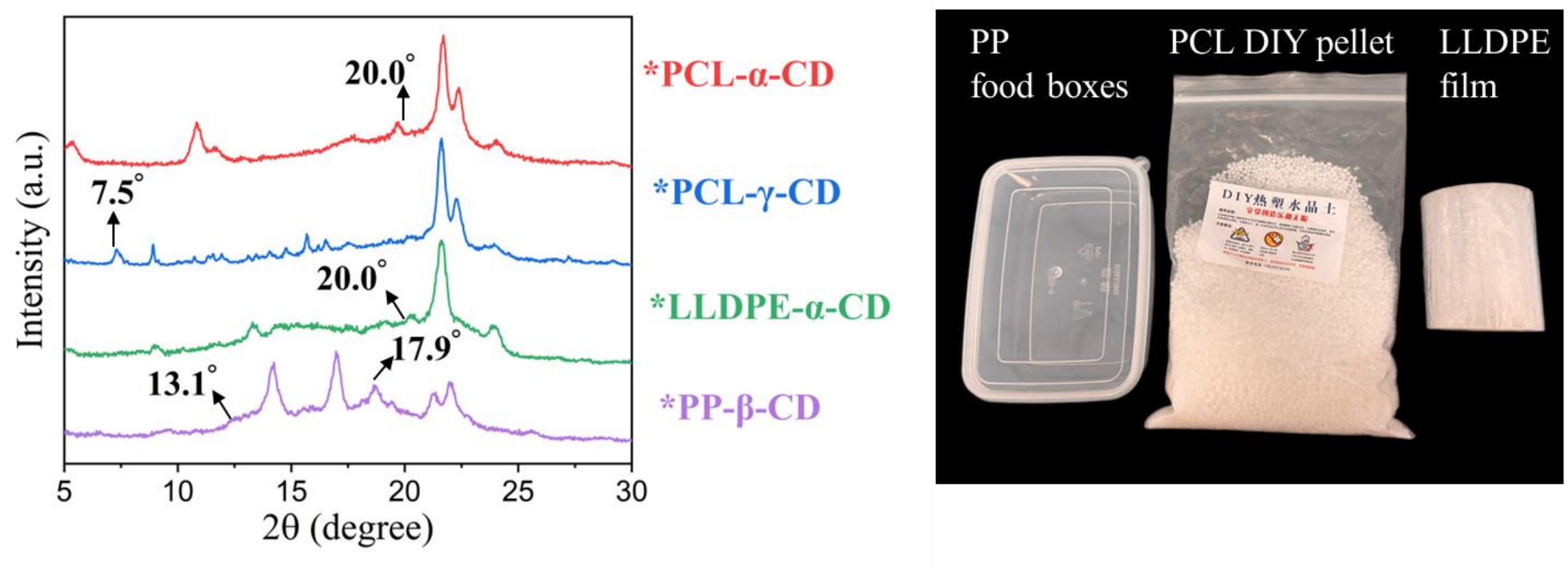

Figure S2. XRD patterns of CD complexes with commercial plastics.

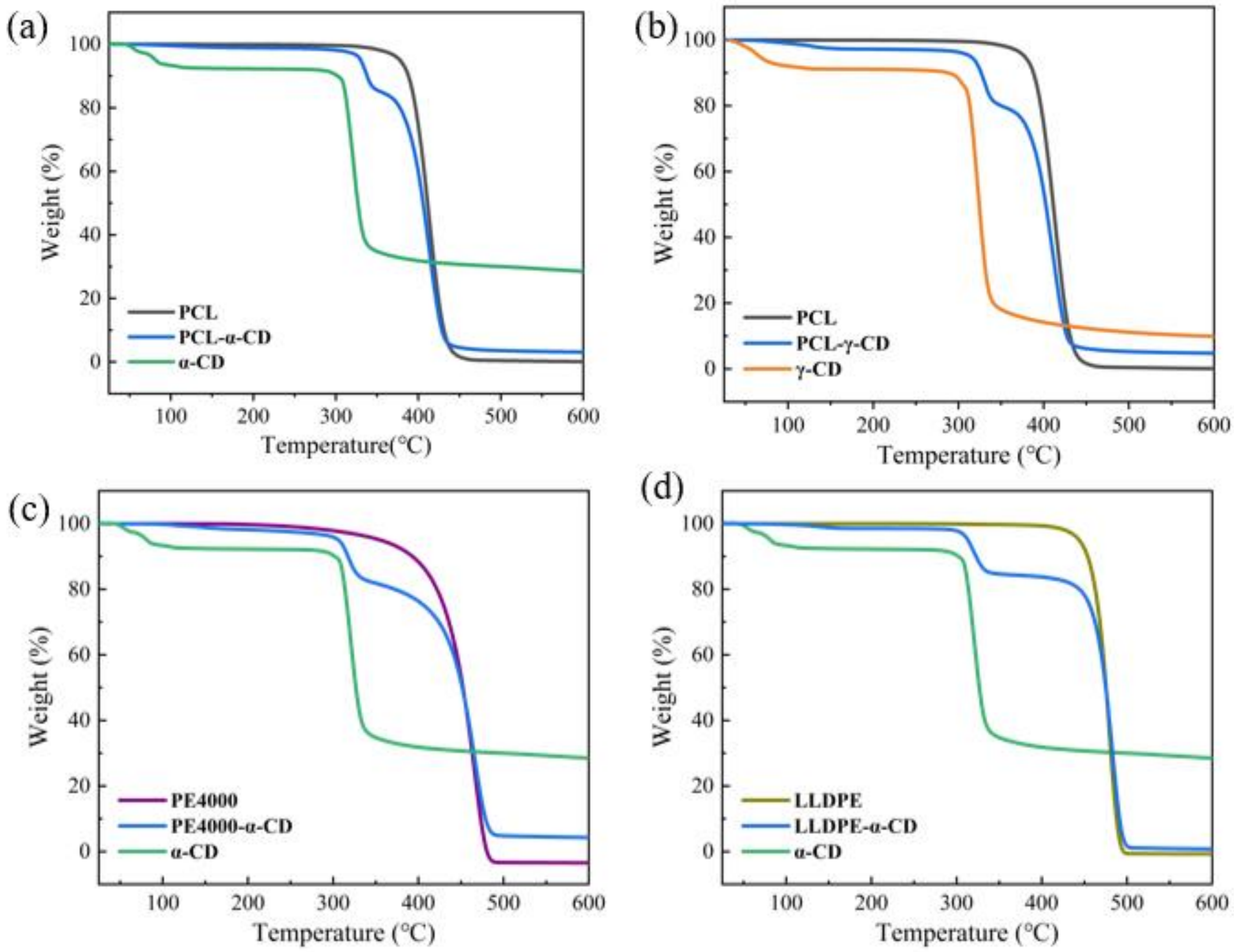

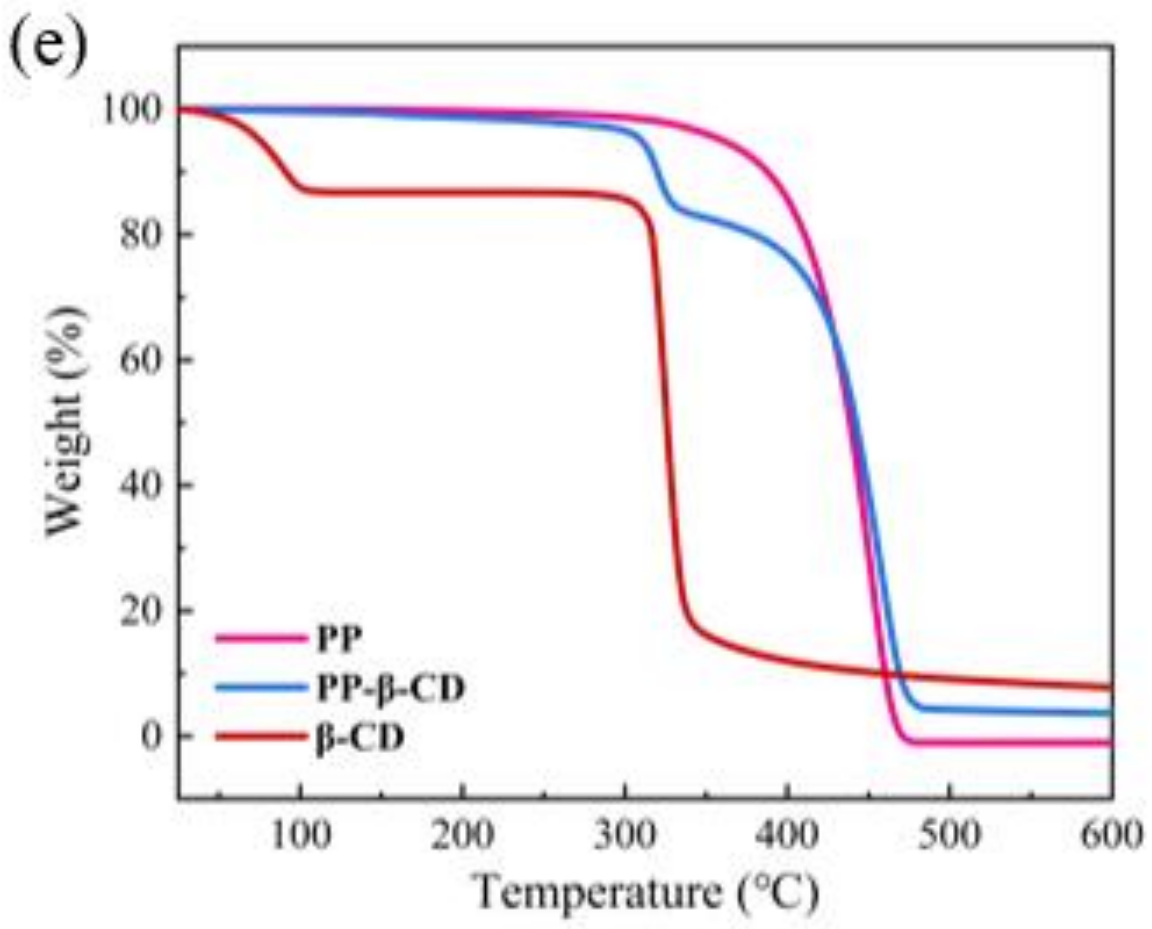

List of decomposition temperatures

	T_d (°C) of free polymer	T_d (°C) of polymer in PICs
PCL	390.2	392.7 (PCL-α-CD)<br>390.8 (PCL-γ-CD)
PE4000	429.7	438.2
LLDPE	462.3	463.4
PP	416.1	426.7

Figure S3. TGA curves of free CDs, free polymers and polymer cyclodextrin inclusion complex.

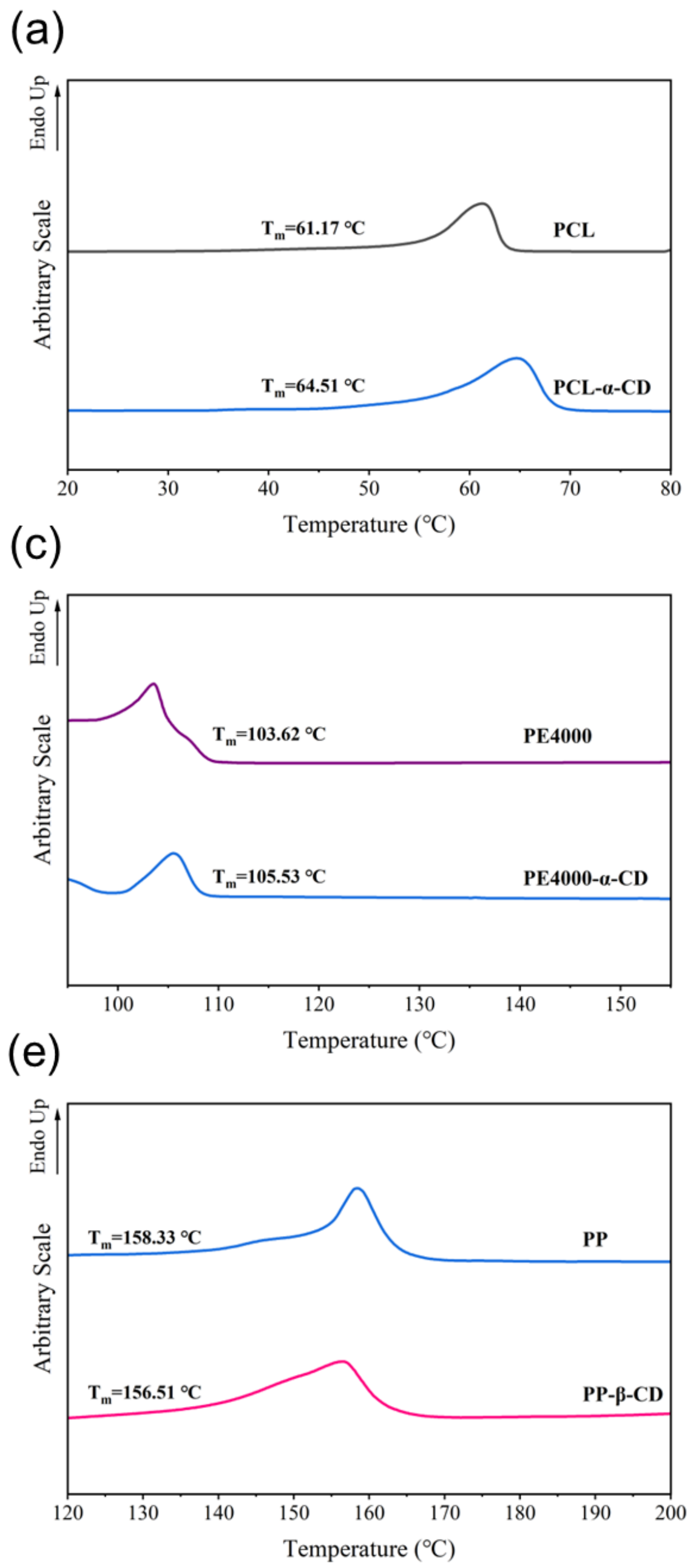

Figure S4. DSC heating scan of free polymers and as-prepared polymer cyclodextrin inclusion complexes.

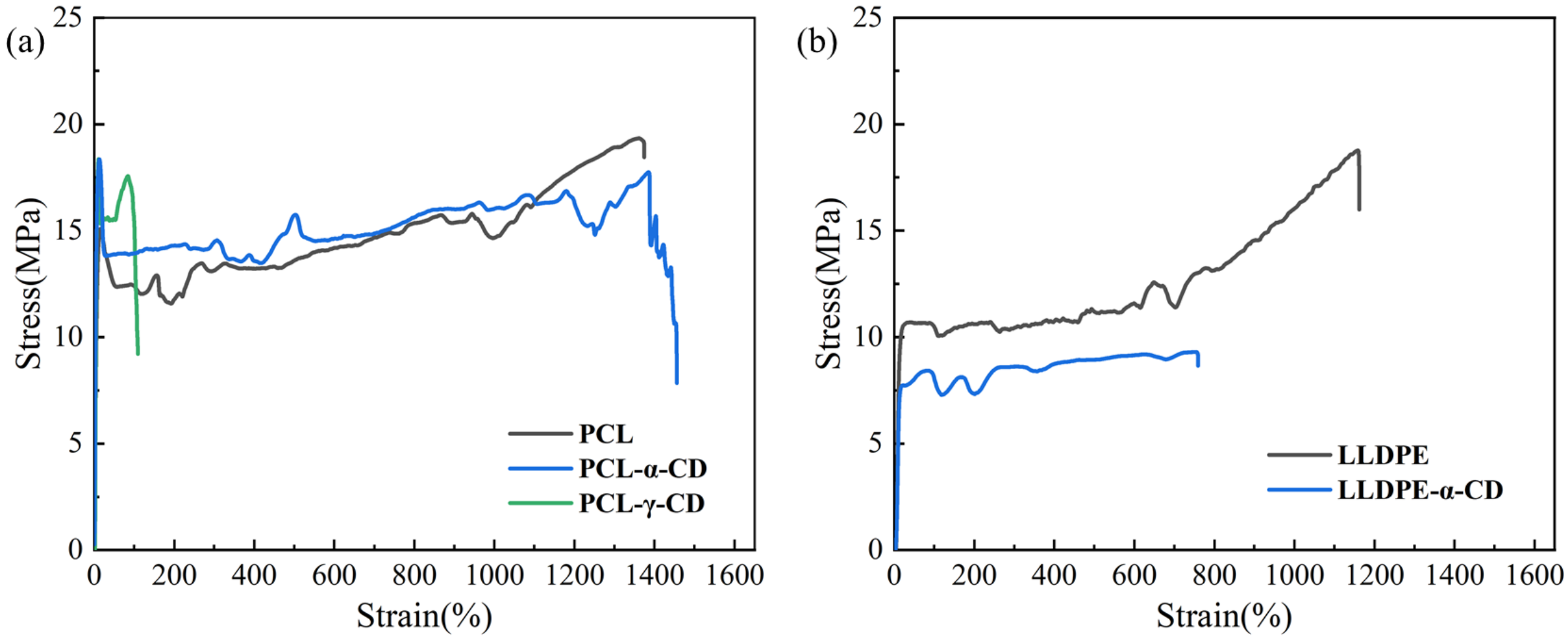

Figure S5. Stress strain curves of free polymers and polymer cyclodextrin inclusion complexes. Other PICs namely PE4000-α-CD and PP-β-CD were too brittle and cannot be shaped into tensile test samples.

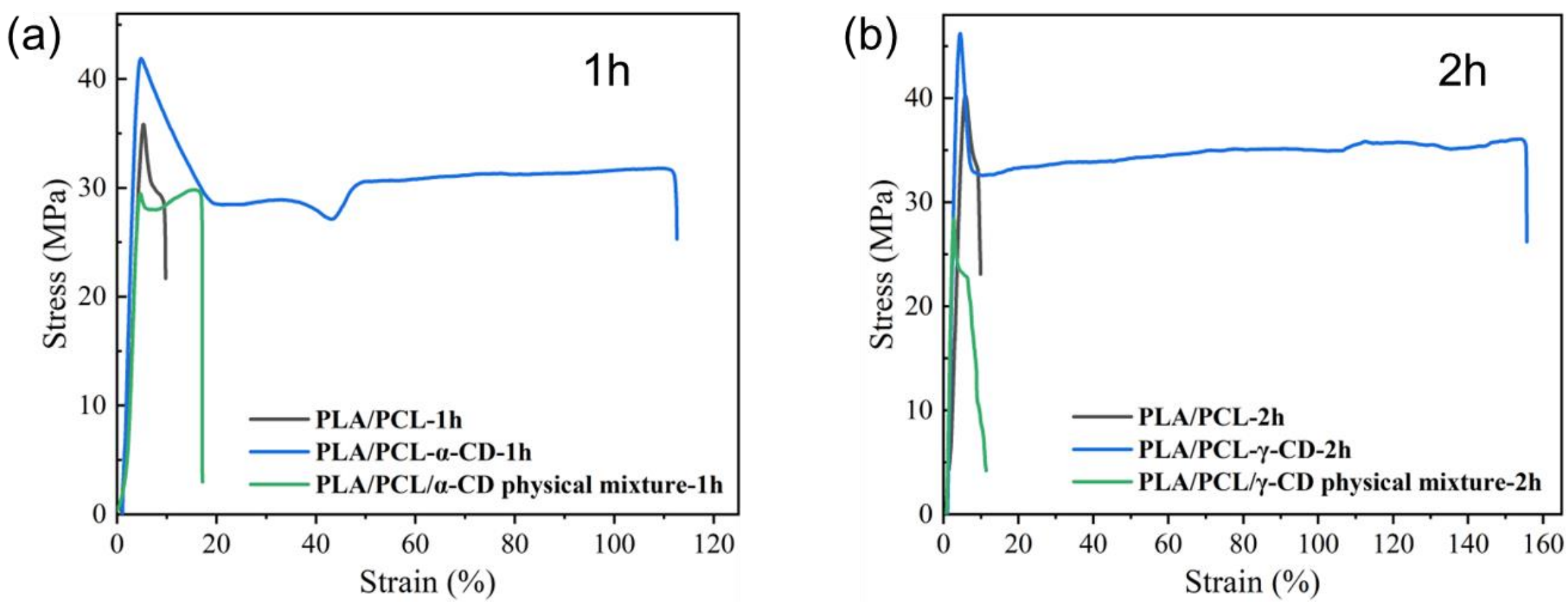

Figure S6. (a) stress strain curves of polymer blends extruded for 1h; (b) stress strain curves of polymer blends extruded for 2h.

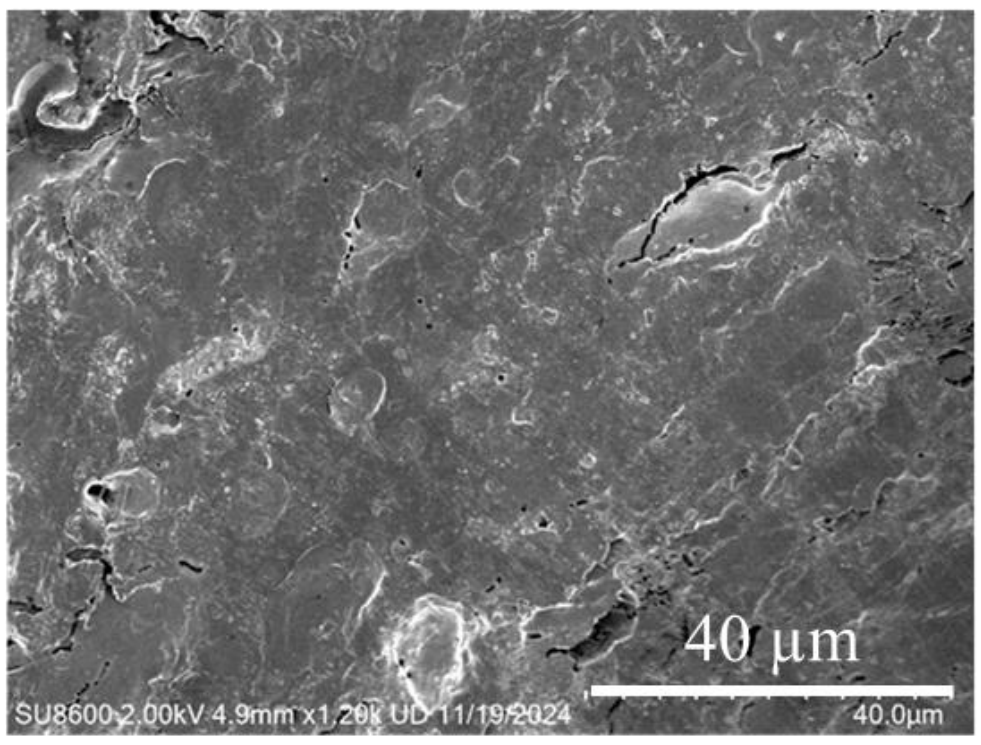

Figure S7. SEM images of PLA/PCL blend for 1h, Scale bar 40 µm.

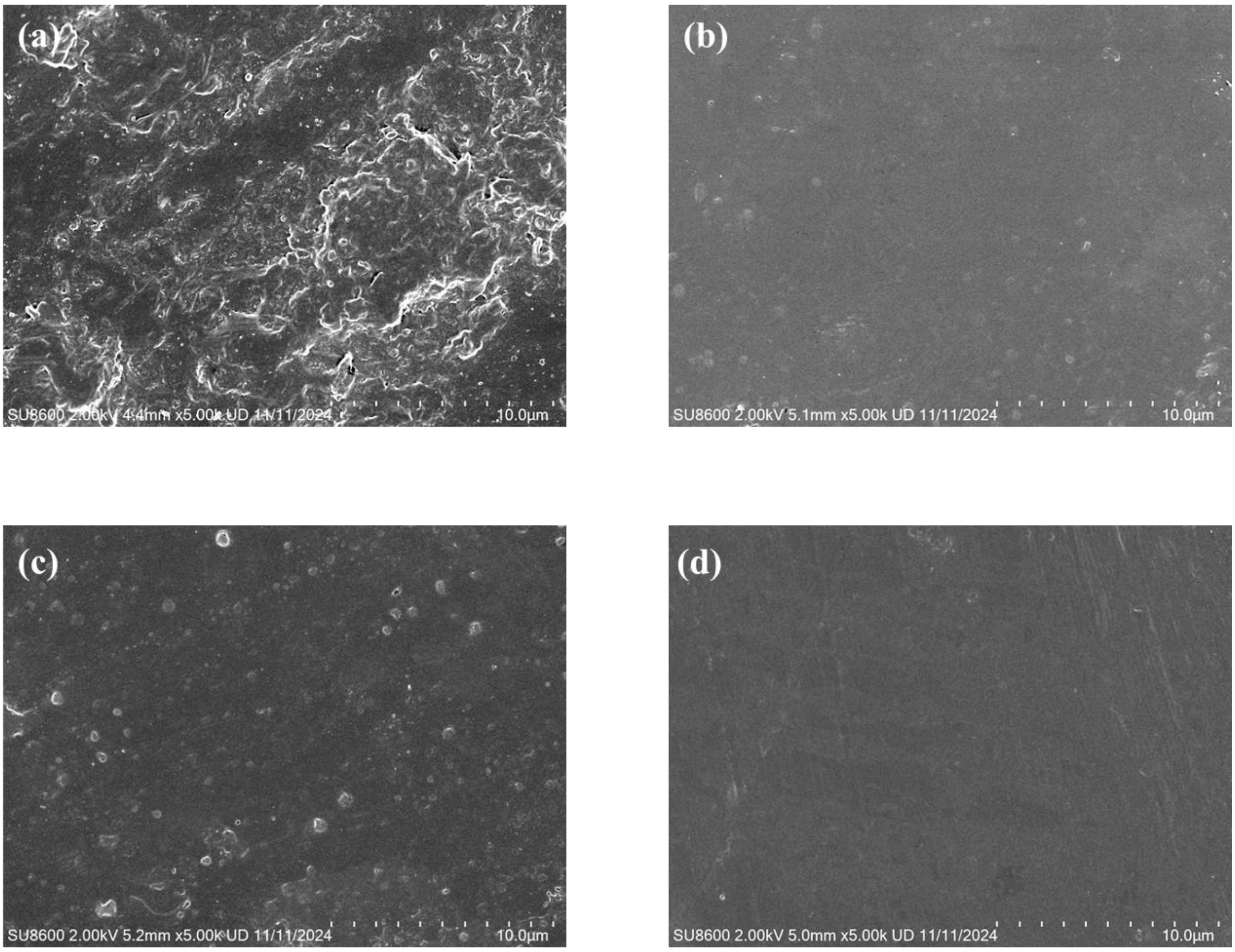

Figure S8. SEM images of (a) PLA/PCL/α-CD physical mixture, (b) PLA/PCL-α-CD blend, (c) PLA/PCL/γ-CD physical mixture, and (d) PLA/PCL-γ-CD blend. Scale bar 10 μm.

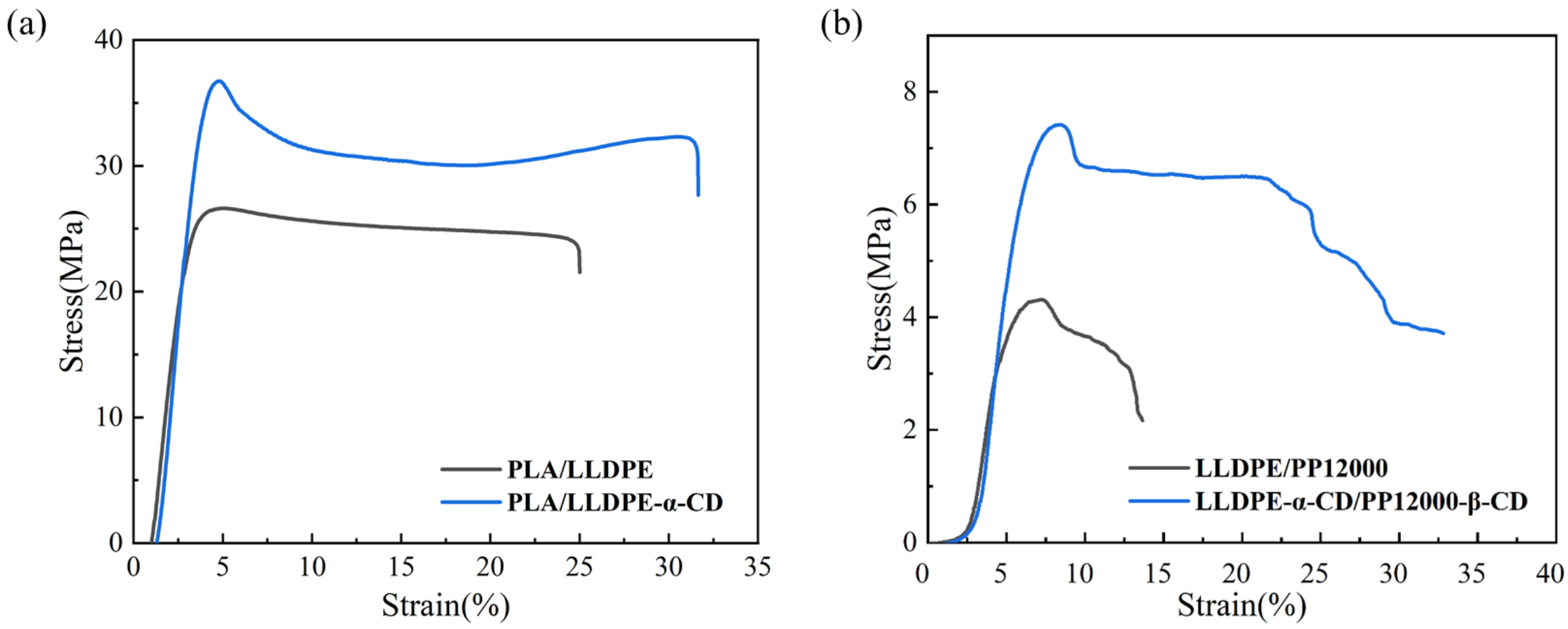

Figure S9. Stress strain curves of (a) PLA/LLDPE blend, PLA/LLDPE-α-CD blend, (b) LLDPE/PP blend, LLDPE-α-CD/PP-β-CD blend.

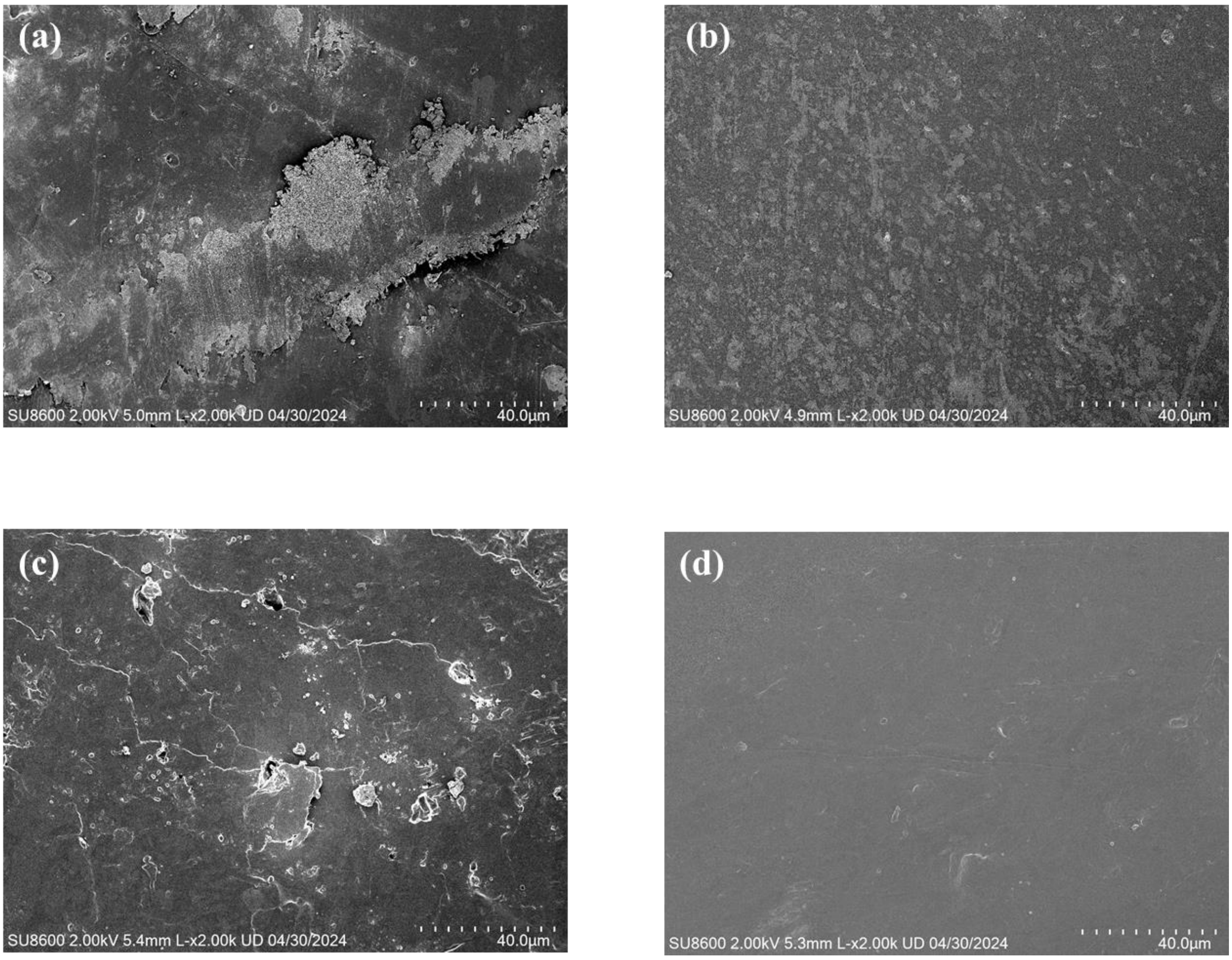

Figure S10. SEM images of (a) PLA/LLDPE blend, (b) PLA/LLDPE-α-CD blend, (c) LLDPE/PP blend, and (d) LLDPE-α-CD/PP-β-CD blend. Scale bar 40 µm.

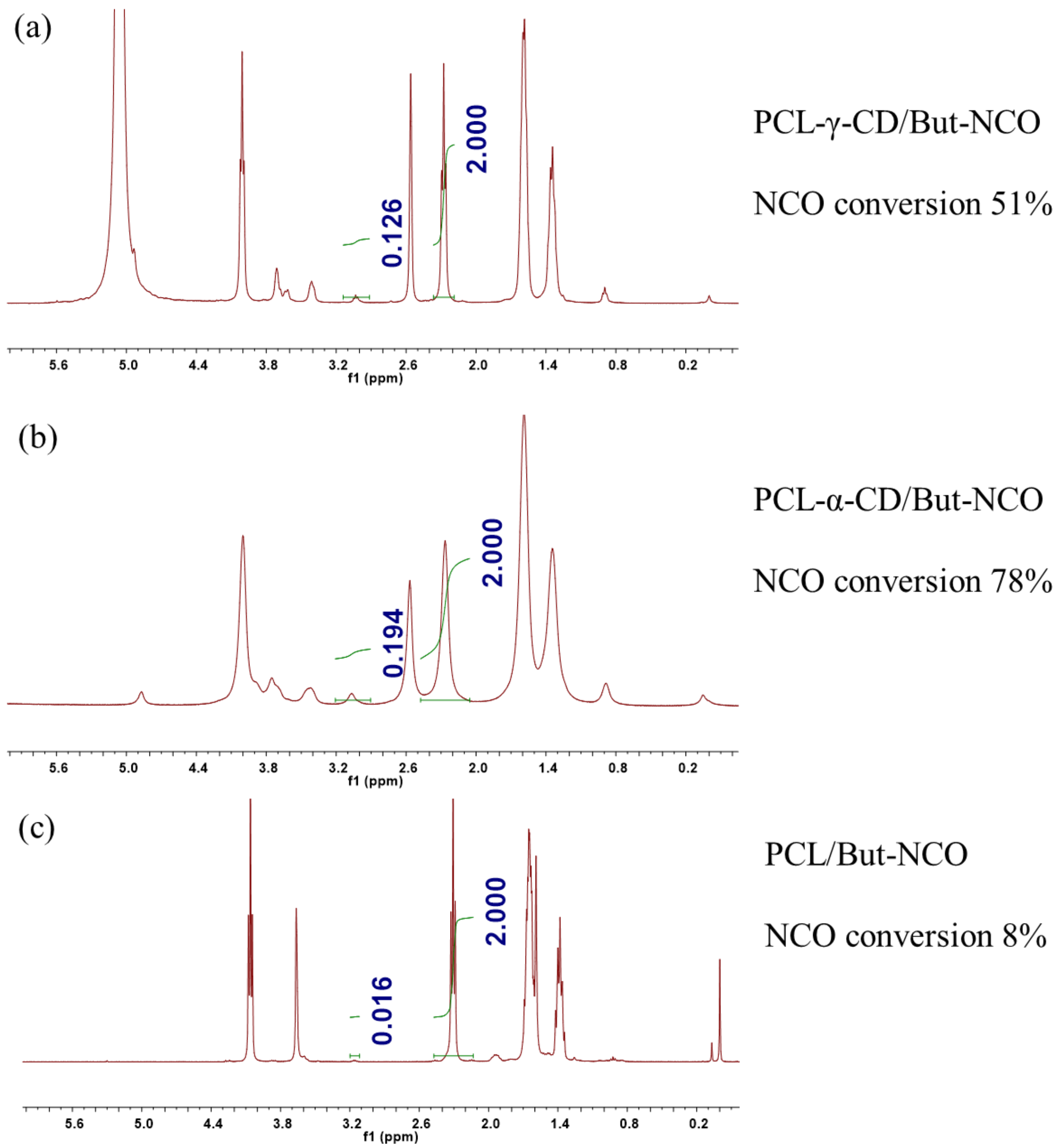

Figure S10. ^{1}H NMR spectra of bulk modification products. Trifluoroacetic acid was added to dissolved PCL-CD/But-NCO products. Conversion ratios were calculated according to the NMR integration and actual amounts of reactants added to the reaction, where chemical shift at 3 ppm is assigned to CH_2 next to the urethane unit of product.
